# Effect of dietary cotton stalk on nitrogen and free gossypol metabolism in sheep

**DOI:** 10.5713/ajas.18.0057

**Published:** 2018-08-27

**Authors:** Halidai Rehemujiang, Aibibula Yimamu, Yong Li Wang

**Affiliations:** 1 College of Grassland and Environmental Science, Xinjiang Agricultural University, Urumqi 830052, China; 2 Xinjiang TianShan Animal Husbandry Bio-engineering CO., LTD, Changji 831100, China

**Keywords:** Cotton Stalk, Nitrogen, Free Gossypol, Metabolism, Sheep

## Abstract

**Objective:**

This study was to investigate the effects of dietary cotton stalk on nitrogen and free gossypol in sheep.

**Methods:**

Treatments included 25% cotton stalk (Treat 1), 50% cotton stalk (Treat 2), and a control (no cotton stalk). Six Xinjiang daolang wethers were cannulated at the rumen and duodenum and fed one of these diets. The effects of these diets on nitrogen and free gossypol absorption and metabolism were determined. Fifteen healthy Xinjiang daolang wethers were assessed for daily gain, tissue lesions, and free gossypol accumulation.

**Results:**

Dry matter intake decreased with increasing dietary cotton stalk. Total tract dry matter digestibility did not significantly differ among treatments. Dietary cotton stalk significantly decreased volatile fatty acids and increased ammonium nitrogen in the rumen. Nitrogen intake was significantly higher in Treat 2 than in the control or Treat 1. Nitrogen retention and free gossypol intake increased with dietary cotton stalk. Duodenal free gossypol flow did not increase, and free gossypol almost disappeared from the rumen. The free gossypol content of plasma and tissue was increased with dietary cotton stalk with liver free gossypol> muscle free gossypol>kidney free gossypol. Elevated dietary free gossypol decreased platelets, hemoglobin, and serum iron. Aspartate aminotransferase and γ-glutamyltransferase increased in Treat 2. With high long-term dietary cotton stalk intake, liver cells were swollen, and their nuclei dissolved. Renal cells were necrotic and the interstitia were enlarged.

**Conclusion:**

With short-term cotton stalk administration, only a small amount of free gossypol is retained in the body. In response to long-term or high free gossypol cotton stalk feeding, however, free gossypol accumulates in, and damages the liver and kidneys.

## INTRODUCTION

Cotton is one of the major crops produced in Xinjiang, China. It is cultivated annually over an area of >1.5 million ha. These large cotton plantations have restricted the production of forage crops in the region. Nevertheless, they provide substantial amounts of by-products like cottonseed meal, cottonseed hulls, and cotton stalk. All of these are used as feed resources. In this region, cotton is raised by high-density dwarfing cultivation. The cotton stubble generated by this method is widely used for wintertime sheep grazing. Some farmers blend the post-harvest cotton stubble and the crushed cotton stalk into the sheep feed. The cotton stalk consists of 22% leaves, 21% bolls, 16% slender stalks, and 41% main stalks (by dry matter [DM]) [1]. The leaves and bolls contain the major nutrients required by grazing ruminants. Cotton stalk contains more crude protein than food crop straw, but it also has relatively higher levels of neutral detergent fiber and acid detergent lignin. Cotton stalk also contains potentially toxic free gossypol. This component has various adverse animal health effects. Halidai et al [2] found that >50% of the free gossypol can be detoxified by granulation or by various chemical or biological means [2]. Sheep consumed 0.50 to 0.70 kg/d crushed cotton stalk and their DM intake was equivalent to 1.3% to 1.8% of their body weight [3].

Although ruminants can detoxify free gossypol, the biochemical mechanism remains unclear [4,5]. Some researchers have found free gossypol may bind proteins with free amino acid sites that bound gossypol may be released as free gossypol and then absorbed during digestion. This reaction impedes free gossypol absorption from the digestive tract [6], and affects liver functions, erythrocyte oxygen-carrying/releasing capacity, respiration rates, feed intake, and reproductive efficiency [5–7]. The absorbed free gossypol is then either retained in the kidneys and muscles or excreted via the feces, urine, or milk [8].

Ruminant feed resources derived from cotton by-products are widely used in Xinjiang. Nevertheless, the absorption, metabolism, and health effects of free gossypol in animals have not yet been reported. The objectives of this study were to determine the effects of various dietary cotton stalk levels on free gossypol metabolism, free gossypol retention, and lesion induction in sheep tissues.

## MATERIALS AND METHODS

### Diets, animals, and sampling

Three dietary treatments were used in this study. The control consisted of 50% wheat straw, 10% corn silage (DM based), and 40% formula concentrate feed. In Treatment (Treat) 1, half the wheat straw was replaced by an equal amount of crushed cotton stalk. For Treat 2, crushed cotton stalk substituted for all the wheat straw. The chemical compositions and components of the experimental diets are shown in Table 1. Six Xinjiang daolang wethers (35.6±4.21 kg) were divided into three groups after being fitted with ruminal and duodenal T-type cannulae. They were assigned to one of three dietary treatments using a 3×3 Latin square design (Exp 1). The sheep were fed twice daily at 0800 h and 1700 h. Chromic dioxide (Cr_2_O_3_) at a concentration of 2 g/kg DM was injected into the rumen via cannula at each feeding time. It was used to estimate duodenal digest flow and fecal output. Fresh water and mineral block (Kang Te Er Feed Technology Co. Ltd., Yue Yang, China) were available *ad libitum* throughout the study.

Each experimental period lasted 21 d with 13 d adaptation and 8 d sampling. Feeds were sampled and recorded daily during the first 4 d of the sampling period. Fecal samples were collected from each sheep. Combined feed, orts, and feces samples were dried at 60°C for 48 h in a forced-air oven, ground to pass through a 1-mm screen, and stored for subsequent analyses. Duodenal digest was sampled over a 2-d period. On the first day, samples were drawn from the duodenal cannulae starting at 0800 h then collected again every 6 h. On the next day, sampling began at 1100 h. One half of the material collected was lyophilized and milled through a 1-mm screen for use in later analyses. The other half was acidified with 50% H_2_SO_4_, frozen at −20°C, and stored for later free gossypol measurements. On the seventh day of the sampling period, rumen fluid was extracted at 0800 h and again at 2000 h. Sample pH was determined immediately upon collection. The rumen fluid was then strained through four layers of gauze, acidified with 50% H_2_SO_4_, frozen at −20°C, and stored until analysis. Blood samples were collected from the jugular vein at the end of experiment and stored in vacutainer tubes. No anticoagulant was added so that the serum could be separated out and its free gossypol content measured.

In the second experiment, 15 healthy Xinjiang daolang wethers were randomly divided into three groups (five sheep per group) and fed for 60 consecutive days *ad libitum* feed containing 10% residue. At the end of the experimental period, three sheep from each group were killed. Muscle, liver and kidney samples were taken for histological observation and determination of tissue free gossypol levels.

Immediately after slaughter, about 2 cm×3 cm of liver and kidney samples, respectively were fixed in 2.5% glutaraldehyde and dehydrated with ethanol, washed with xylene and paraffin embedded. The paraffin embedded tissue blocks were sectioned with a microtome, and the sections placed on a glass slide and stained with stained with hematoxylin and eosin (HE). The slides were sealed with neutral rubber and examined with an Olympus BH-2 optical microscope (Olympus Corp., Shinjuku, Tokyo, Japan).

Another 1 mm×1 mm×1 mm of liver and kidney samples were fixed at 4°C in 2.5% glutaraldehyde. Then the tissue blocks were cropped and fixed in 1% osmium tetroxide, dehydrated in an acetone gradient, embedded in epoxy resin (#812) and electronic dyed with uranyl acetate. Ultrathin sections were made with a DIATOME ultra 45° and examined in a Nissan JEOL TEM 1230 transmission electron microscope (JEOL, Tokyo, Japan).

### Chemical analyses

Samples of feed, duodenal digest, and feces were analyzed for crude protein [9]. Natural detergent fiber and acid detergent fiber were analyzed according to the procedures described by Van Soest et al [9]. Calcium and phosphorus were determined by the methods described by AOAC [10]. The chromium concentrations of the duodenal digest and the feces were measured by colorimetry [11]. The free gossypol content in the rumen fluid, duodenal digest, feces, blood, and tissue were determined according to the method of Mena et al [12].

Volatile fatty acid (VFA) composition of the rumen fluid was determined by high-performance liquid chromatography fitted with flame ionization detector detection and a capillary column (C18, 150×4.6 mm, 1 mL/min, 210 nm).

At the start of the trial and every 15 d thereafter, the lambs were weighed before- and after feeding, and the weight gain was calculated.

Tissue samples from all major organ systems were fixed in 10% v/v neutral buffered formalin. They were prepared for histological examination by routine paraffin embedding. Sections (6-μm thick) were made and stained with HE. They were examined under an Olympus BH-2 optical microscope (Olympus Corp., Shinjuku, Tokyo, Japan) and an EM-400 electron microscope (Koninklijke Philips N.V., Amsterdam, The Netherlands).

### Calculations and statistical analysis

The data for DM, free gossypol, and nitrogen intake, duodenal flow, and apparent digestibility were all reported as least square means±standard error of the mean. They were analyzed as a Latin square with a factorial treatment arrangement using SPSS v. 17.0 (IBM Corp., Armonk, NY, USA). Overall differences in treatment means were considered significant when p<0.05.

## RESULTS

### Dry matter intake and digestion

Feed intake increased with compositional percentage of cotton stalk supplementation (p<0.01) (Table 2). The DM digestibility in the rumen and post-rumen significantly differed among treatments. The DM excretion into the feces decreased with increasing proportion of cotton stalk. There were no significant differences in total tract DM digestibility among treatments.

### Rumen fermentation quality

For all animals tested, the rumen fluid pH was in the normal range of 5.93 to 6.13. After various proportions of cotton stalk were added to the diet, the ammonium nitrogen content in the rumen fluid changed to varying degrees. The rumen ammonium level in Treat 1 was significantly lower than those for the control and Treat 2 (p<0.01). The acetic acid content in the experimental groups was significantly lower than that of the control group (p<0.05). There were significant differences in total VFAs or propionic acid concentrations among the groups (p>0.05). The ratio of acetic acid to propionic acid decreased with increasing cotton stalk content in the diet (Table 3).

### Nitrogen intake and balance

Nitrogen intake, digestion, and retention are shown in Table 4. Nitrogen intake was significantly higher in Treat 2 than the other treatments (p<0.05). Fecal nitrogen excretion did not significantly differ among treatments (p>0.05). Nitrogen retention increased with the proportion of cotton stalk in the diet (p<0.05).

### Free gossypol intake and digestion

Dietary free gossypol intake and digestion, including free gossypol its disappearance and digestibility in the rumen and post-rumen did not significantly differ among treatments (Table 5). Free gossypol intake increased with the proportion of cotton stalk in the diet. Free gossypol excretion in the feces and urine increase with gossypol intake (p<0.05). The almost of intake free gossypol disappeared in the rumen. There were no significant differences among treatments in terms of the amount of free gossypol disappearing from the post-rumen (p>0.05). Free gossypol retention was significantly higher in Treat 2 than in either the control or Treat 1.

### Growth performance

The mean body weight and daily body weight gains for the 60-d study are presented in Table 6. There were significant differences in body weight gain and average daily gain among treatments.

### Blood composition

Relative to the control group, the platelet counts, hemoglobin levels, and Fe content were significantly lower in Treat 1 and Treat 2 (p<0.05). For the other blood components, however, no significant differences among treatments were detected (p>0.05). Serum aspartate aminotransferase and γ-glutamyltransferase were significantly higher in Treat 2 than the others (p<0.05) (Table 7).

### Tissue free gossypol levels

Plasma gossypol concentrations significantly increased (p<0.05) with supplemental cotton stalk levels. Free gossypol residue levels in the liver, kidney, and muscle significantly differed among treatments. Free gossypol tissue retention was higher in Treat 2 than in either the control or Treat 1. Free gossypol residues very significantly increased in the liver with free gossypol intake (Table 8).

### Microstructural changes in the liver and kidney

**Figure 1:**
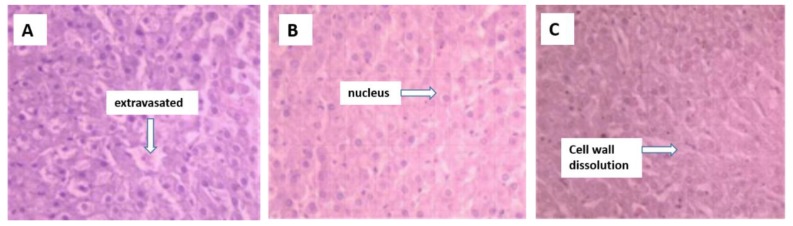
Hematoxylin and eosin staining of the liver from sheep fed control (A), 25% cotton stalk (B), and 50% cotton stalk (C) (10×40 objective view).

Hepatocyte swelling and nuclear dissolution, central venous- and portal vein congestion, local tissue necrosis, and inflammatory cell infiltration in necrotic areas were observed in the livers of the sheep ingesting cotton stalk (Figures 1A–1C).

**Figure 2:**
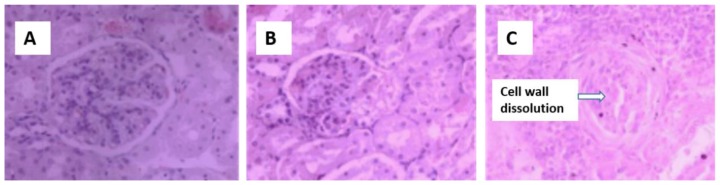
Hematoxylin and eosin staining of the kidney from sheep fed control (A), 25% cotton stalk (B), and 50% cotton stalk (C) (10×40 objective view).

**Figure 3:**
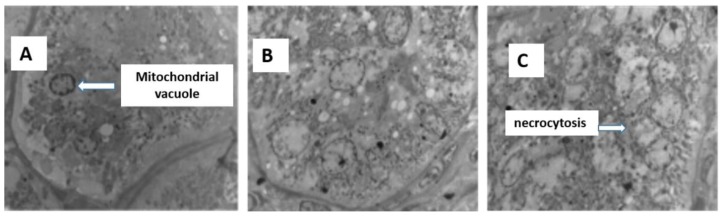
Ultrastructure of the liver from sheep fed control (A), 25% cotton stalk (B), and 50% cotton stalk (C).

Control kidneys (Figure 2A) have normal glomerular morphology and no swelling in the glomerular capillary network. Diseased kidneys have relatively smaller glomerular volumes (Figures 2B–2C), capillary network atrophy, thickening of the glomerular basement membrane, necrosis of the splenic cells in the renal tubules, and enlargement of the renal interstitium. Kidney ultrastructure (Figures 3D–3F) shows changes in the podocytes, vacuolar necrosis, swelling of the internal structures of the glomeruli and secondary structures, shrinkage of the endothelial cells, and mitochondrial swelling.

## DISCUSSION

In this study, various levels of dietary cotton stalk were administered to sheep to determine their effects on free gossypol metabolism, retention, and lesion induction in different tissues. Van Soest [9] concluded that feed intake depends on cell wall content. In cotton stalk, the crude protein content is high, but the lignin content is 2 to 3 times greater than it is in other crop straws. Owing to high cellulose and lignin levels in cotton stalk, DM intake varied inversely with dietary cotton stalk consumption (p<0.05). Some studies have found that cotton stalk limited feed intake in sheep. Wei et al [1] reported that sheep being fed crushed cotton stalk voluntary ingested 0.693 kg/d. We observed that sheep being given concentrate and corn silage would consume 0.63±0.05 kg/d when crushed cotton stalk was blended with the other food [13].

Gossypol is a naturally occurring toxin produced by pigment glands throughout the cotton plant. It occurs in both free and bound forms. The free gossypol in feed is toxic to animals. Free gossypol intake varies directly and linearly with dietary cotton stalk consumption. In this study, free gossypol intake reached 322.20 mg and 480.31 mg in Treats 1 and 2, respectively. Nevertheless, there were no significant differences among treatments in terms of duodenal free gossypol flow and post-rumen free gossypol disappearance (p>0.05). Excretion in the feces and urine accounted for 5% and 40% of the free gossypol clearance, respectively. Some researchers [12] have found free gossypol may bind proteins with free amino acid sites or iron ions (Fe^3+^) that bound gossypol may be released as free gossypol and then absorbed during digestion. In this study, despite the difference in free gossypol intake, the free gossypol inflow to the duodenum did not differ between the treatments and most free gossypol disappeared in the rumen. The proportions of free gossypol absorbed in the post-rumen following free gossypol intake were only 5.48% and 4.73% in Treats 1 and 2, respectively.

Liu et al [14] feeding sheep containing >200 mg/kg fee gossypol diet significantly reduces their weight gain. In this study, the Treat 2 diet, which contained >200 mg/kg free gossypol, adversely affected growth performance by reducing daily weight gain. Free gossypol might accumulate in animal tissues over time and have a negative impact on growth performance. Free gossypol toxicity is associated with alterations in blood parameters [15,16]. Gossypol may combine with iron, inhibit the synthesis of respiratory enzymes, and generate excessive reactive oxygen species in the mitochondria [17]. In the present study, the serum aspartate aminotransferase and γ-glutamyltransferase content linearly increased (r^2^ = 0.84, p = 0.01; r^2^ = 0.99, p = 0.03), while the iron content linearly decreased (r^2^ = 0.99, p = 0.04) in response to cotton stalk ingestion. Elevated aspartate aminotransferase and γ-glutamyltransferase activities were correlated with liver metabolism impairment. Gossypol concentrations in the plasma and liver increased with dietary cotton stalk intake. Free gossypol retention in the liver, kidney, and muscle significantly differed among treatments; liver free gossypol>muscle free gossypol>kidney free gossypol. Tissue free gossypol accumulation caused liver cell swelling and nuclear dissolution, renal- and renal tubular spleen cell necrosis, and renal interstitial enlargement.

In this study, although the digestion and metabolism of free gossypol and the residue of body tissue were identified, there is still a need to continue to study and explain the effects of other residues such as pesticide, chlormequat chloride in cotton stalk on the healthy breeding of sheep.

## Figures and Tables

**Table 1:** Ingredient and chemical composition of basal diet1) (dry matter based)

Factor	Dietary composition		

	Control	25% cotton stalk	50% cotton stalk
Constituent (%)			
Wheat straw	50.0	25.0	0.0
CS	0.0	25.0	50.0
Corn silage	10.0	10.0	10.0
Concentrate	40.0	40.0	40.0
Total	100.0	100.0	100.0
Nutrient composition (%)			
Crude protein	10.12	10.89	11.54
Ether extract	4.12	3.82	3.53
NDF	39.81	39.35	36.95
ADF	24.04	27.7	28.25
Ca	0.31	0.43	0.55
P	0.24	0.25	0.27
FG (mg/kg)	81.36	197.26	253.56

CS, cotton stalk; NDF, neutral detergent fiber; ADF, acid fetergent giber; Ca, calcium; P, phosphorus; FG, free gossypol.

1) Concentrations of gossypol in all diets were calculated based on the gossypol content of the cotton products and their inclusion rates in the respective diets.

**Table 2:** Dry matter intake and digestibility based on dry metter content by sheep fed dietary cotton stalk

Factor	Treatment			SEM	p value

	Control	25% cotton stalk	50% cotton stalk		
Intake (kg/d)	1.659±0.21^a^	1.634±0.04^a^	1.534±0.33^b^	0.06	0.02
Duodenal flow (kg/d)	0.968±0.12	1.116±0.05	1.086±0.09	0.01	0.26
Fecal output (kg/d)	0.732±0.07	0.706±0.07	0.681±0.05	0.05	0.74
Disappearance (kg/d)					
Rumen	0.690±0.14^b^	0.520±0.04^a^	0.445±0.02^a^	0.02	0.13
Post-ruminal	0.236±0.06^a^	0.410±0.02^b^	0.406±0.04^b^	0.05	0.04
Total digestive tract	0.927±0.08	0.927±0.07	0.853±0.02	0.04	0.50
Digestibility (%)					
Rumen	41.60±7.99^a^	31.68±2.80^b^	29.15±0.91^b^	0.87	0.03
Post-ruminal	14.28±3.49^b^	25.09±1.44^a^	26.47±3.01^a^	3.71	0.04
Total digestive tract	55.88±4.50	56.77±4.24	55.62±2.14	3.00	0.95

SEM, standard error of the mean.

ab Values with different lowercase-within the same row are significantly different (p<0.05).

**Table 3:** Rumen fermentation quality

Parameter	Treatment			SEM	p value

	Control	25% cotton stalk	50% cotton stalk		
pH	6.02±0.17	6.04±0.01	5.93±0.01	0.03	0.19
Acetic acid (A) (mM)	106.22±3.96^a^	85.46±9.68^b^	80.50±0.26^b^	3.06	0.03
Propionic acid (P) (mM)	54.70±1.52^a^	53.99±4.92^a^	64.10±1.54^b^	7.46	0.05
Total acid (mM)	165.23±9.91^a^	148.61±9.51^b^	144.60±1.79^c^	6.98	0.03
A/P	1.80±0.06^a^	1.36±0.29^b^	0.95±0.29^c^	0.08	0.05
Ammonia nitrogen (mg/dL)	10.99±1.01^a^	11.20±0.75^b^	16.37±0.38^c^	2.18	0.01

SEM, standard error of the mean.

a–c Values with different lowercase-letter superscripts are significantly different (p<0.05).

**Table 4:** N intake and digestibility based on dry matter

Factor	Treatment			SEM	p value

	Control	25% cotton stalk	50% cotton stalk		
N intake (g/d)	186.16±9.44^c^	201.12±6.26^b^	215.32±6.51^a^	5.54	0.01
Nitrogen excretion (g/d)					
Fecal	54.09±3.01	57.38±0.51	55.89±4.28	1.67	0.75
Urinary	18.29±0.63^c^	16.38±0.66^b^	21.36±0.25^a^	0.50	0.02
Apparent N digestibility (%)	70.77±2.75	70.29±2.39	73.43±0.19	1.52	0.06
Nitrogen retention					
g/d	113.78±10.96^c^	127.36±3.93^b^	138.07±9.07^a^	6.38	0.01
% of N intake	61.12±2.88	63.33±0.66	64.12±0.58	1.62	0.02
% of absorbed N	86.15±0.85	88.61±0.41	86.60±1.58	0.52	0.06

SEM, standard error of the mean.

a–c Values with different lowercase-letter superscripts are significantly different (p<0.05).

**Table 5:** Free gossypol intake and digestibility (based on dry matter)

Factor	Treatment			SEM	p value

	Control	25% cotton stalk	50% cotton stalk		
Intake (mg/d)	134.93±1.68^c^	322.20±0.66^b^	480.31±10.50^a^	0.06	0.01
Duodenal flow (mg/d)	25.48±1.58^c^	31.73±0.42^b^	41.95±3.59^a^	1.25	0.02
FG excretion (mg/d)					
Fecal	6.99±1.19^c^	14.08±0.19^b^	19.76±2.63^a^	1.31	0.03
Urinary	83.75±4.01^c^	110.96±6.41^b^	207.73±38.51^a^	12.90	0.02
Disappearance (mg/d)					
Ruminal	108.24±3.27^a^	290.47±0.23^b^	428.36±6.91^a^	4.94	0.01
Post-ruminal	19.70±0.38	17.65±0.04	22.78±6.21	1.48	0.45
Digestibility (%)					
Ruminal	80.21±1.01^b^	90.15±0.08^a^	91.28±0.39^a^	0.50	0.02
Post-ruminal	14.60±0.33^c^	5.48±0.02^b^	4.73±0.84^a^	0.82	0.01
Total digestive tract	94.81±0.67	95.64±0.10	96.01±0.45	0.44	0.32

SEM, standard error of the mean; FG, free gossypol.

a–c Values with different lowercase-letter superscripts are significantly different (p<0.05).

**Table 6:** Sheep growth performance

Parameter	Treatment			SEM	p value

	Control	25% cotton stalk	50% cotton stalk		
Initial body weight	36.00±2.16	37.00±1.38	36.7±4.80	6.33	0.93
Final body weight	47.52±1.72	45.66±1.68	43.94±1.82	7.08	0.39
Total body weight gain	11.51±1.56^a^	11.03±1.12^a^	7.20±0.87^b^	3.31	0.03
ADG (kg/d)	0.19±0.02^a^	0.15±0.01^b^	0.12±0.01^c^	0.01	0.02

SEM, standard error of the mean; ADG, average daily gain.

a–c Values with different lowercase-letter superscripts are significantly different (p<0.05).

**Table 7:** Sheep blood chemistry

Factor	Treatment			SEM	p value

	Control	25% cotton stalk	50% cotton stalk		
White cell count (10^9^/L)	5.48±0.95	6.29±0.49	5.48±0.40	0.26	0.41
Red cell count (10^12^/L)	4.08±0.68	5.85±2.09	7.12±0.92	0.45	0.14
Hemoglobin concentration (g/L)	113.50±3.54	114.67±13.87	107.67±13.87	2.77	0.55
Platelets (10^9^/L)	254.50±14.85^b^	225.67±104.7^c^	182.00±2.65^a^	24.25	0.03
ALB (g/L)	34.00±1.00	33.00±1.00	35.00±2.65	0.56	0.42
GLU (mM/L)	3.23±0.37	3.90±0.10	4.03±0.12	0.11	0.01
BUN (mM/L)	3.23±0.37^c^	4.68±0.92^a^	3.64±0.47^b^	0.23	0.04
CHO (mM/L)	1.62±0.21^b^	1.86±0.33^ab^	2.39±0.63^a^	0.11	0.03
TG (mM/L)	0.25±0.09	0.37±0.01	0.29±0.09	0.02	0.21
AST (U/L)	106.00±8.74	113.00±3.61	145.67±2.73	9.96	0.01
GGT (U/L)	47.50±1.50^b^	55.50±1.52^a^	61.01±2.10^a^	5.16	0.03
Mg (mg/dL)	0.93±0.01	0.94±0.01	0.92±0.02	0.01	0.06
Ca (mM/L)	2.71±0.08^ab^	2.70±0.08^ab^	2.52±0.22^a^	0.03	0.02
P (mM/L)	1.81±0.27	2.10±0.18	2.16±0.76	0.11	0.09
Fe (μM/L)	29.23±2.38^a^	26.45±1.65^b^	23.31±1.79^c^	1.03	0.04

SEM, standard error of the mean; ALB, albumin; GLU, glucose; BUN, blood urea nitrogen; CHO, cholesterol; TG, triglyceride; AST, aspartate aminotransferase; GGT, γ-glutamyl transpeptidase.

a–c Values with different lowercase-letter superscripts are significantly different (p<0.05).

**Table 8:** Free gossypol retention in fattening sheep body tissues (dry matter; mg/kg)

Tissue	Control	25% cotton stalk	50% cotton stalk	SEM	p value
Blood	98.05±0.76^a^	118.66±2.16^b^	121.13±1.84^b^	0.44	0.03
Liver	0.72±0.02^a^	4.88±0.02^b^	12.27±0.03^c^	0.02	0.02
Kidney	0.93±0.02^a^	1.88±0.03^b^	2.15±0.02^c^	0.05	0.01
Muscle	1.56±0.02^a^	1.84±0.02^b^	2.57±0.01^c^	0.03	0.01

SEM, standard error of the mean.

a–c Values with different lowercase-letter superscripts are significantly different (p<0.05).
